# Relaxant effect of *Humulus lupulus* extracts on isotonic rat's ileum contractions

**Published:** 2014

**Authors:** Seyed Hassan Hejazian, Seyyed Majid Bagheri, Mohammad Hossein Dashti-R

**Affiliations:** 1 *Department of Physiology/herbal Medicine Research Center, Shahid Sadoghi University of Medical Sciences, Yazd, I. R. Iran *

**Keywords:** *Acetylcholine*, *Antispasmodic*, *Humulus lupulus*, *Ileum*

## Abstract

**Objective:** Many biological studies have been done to determine the activity of medicinal plants on gastrointestinal function. Since acetylcholine is the major transmitter involved in the gastrointestinal motility and there are some evidences regarding the cholinergic modulatory effect of hops extract, in the present study spasmolytic and antispasmodic action of hops (*Humulus lupulus*) on acetylcholine-induced contraction in isolated rat's ileum was evaluated.

**Material and Methods:** In this study, pieces of isolated rat's ileum were mounted in the internal chamber of an organ bath which was filled with Tyrode’s solution and tightly tied to the lever of an isotonic transducer. The contractile responses were recorded by using an oscillograph device. In the presence of normal saline and different concentrations of hops (0.1, 0.3, and 0.5 mg/ml), the amplitude of contractions induced by10^-12 ^up to 10^-2 ^M acetylcholine was determined. The spasmolytic action of the same extract concentrations was also examined on contraction induced by 10^-4^ acetylcholine.

**Results: **Our findings indicate that hops extract reduces acetylcholine-induced contraction in all concentrations. The significant inhibitory effects of 0.1, 0.3, and 0.5 mg/ml hops extract on contraction induced by 10^-3 ^M acetylcholine were 81.9, 77, and 29.3, respectively (p<0.05).

**Conclusion:** According to our findings, hops extract poses a potent spasmolytic and antispasmodic action on acetylcholine-induced contraction in isolated rat’s ileum which may be mediated by cholinergic systems.

## Introduction

*Humulus* *lupulus* (*H. lupulus*) is a medicinal herb wildly grown in Europe, Asia, and North America (Reeves & Richards, 2011 ▶). In traditional medicine, hop is most often used to treat the symptoms of anxiety such as nervousness, overexcitability, restlessness, and insomnia (Viesti et al., 2011 ▶). The anti-anxiety, analgesic, and hypnotic effects of hops have also been mentioned in Iranian herbal medicine pharmacopoeia (Qasemi, 2003 ▶). Hops are composed of flavonoids, phenolic acids, and terpenophenolics (Kavalier et al., 2011 ▶). 

The most important hop flavonoids are prenylflavonoids and xanthohumol (Chen et al., 2012 ▶). Alpha acids have been mentioned to be the major component of hop flower which enhance the pentobarbital effect and exhibit antidepressant effect (Zanoli et al., 2005 ▶**)**. Other pharmacologically active components of hop are alpha and beta bitter acids (Qasemi, 2003 ▶). The main component of alpha-bitter acids is humulone and for beta-bitter acids the main components are lupulone, colupulone, and adlupulone (Qasemi, 2003 ▶). Several therapeutic effects including analgesic (Hejazian et al., 2007 ▶)^,^ sedative (Hansel et al., 1980 ▶; Muller-Limmroth & Ehrenstein, 1997 ▶; Dimpfel et al., 2008 ▶), and estrogenic (Chadwick et al., 2006 ▶) have been demonstrated for hop. It has been suggested that hop exerts its sedative, hypnotic, and analgesic action via inhibition of central nervous cholinergic system (Dimpfel et al., 2006 ▶).

It also increases the gastric secretions by stimulating the enteric cholinergic neurons (Kurasawa et al., 2005 ▶). Since the acetylcholine is a major stimulatory transmitter for gastrointestinal motility and due to the controversial suggestions about the effect of hops extract on cholinergic system, this study was done to evaluate the spasmolytic and antispasmodic effect of hops extract on acetylcholine-induced contraction in isolated rat's ileum.

## Material and Methods

**Animals**

For this study, 36 male adult Wistar rats weighting 200-250 gr were collected from the animal house in Shahid Sadoughi University of Medical Sciences (Yazd, Iran). For experimental procedures, permission of the Animal Ethics Committee of Shahid Sadughi Medical Science University (Yazd, Iran), in accordance with the internationally accepted principles for laboratory animal use and care mentioned by the European Community guidelines was obtained.

**Plant extraction**

The plant material was identified by a botanist in the herbarium of Yazd Herbal Medicine Research Center. Twenty grams of air-dried female flowers (cones) of *Humulus Lupulus* L. were gently grounded and macerated in 200 ml of double distilled water vibrated for 24 h. The mixture was then filtered and the yielded solution was dried under the room temperature. The concentrations of 0.1, 0.3, and 0.5 mg/ml were prepared by dissolving the dried extract in double distilled water.

**Tissue preparation and experimental procedure**

The method used in this experiment was according to our previous studies (Hejazian et al., 2009 ▶; Hejazian et al., 2011 ▶). Briefly, animals were deeply anesthetized by 75 mg/kg ketamin and 25 mg/kg xilazin. Following incision through the abdominal wall, pieces of ileum (2 cm in length) were isolated and mounted in the internal chamber of an organ bath which was filled with Tyrode’s solution and tightly tied to the lever of an isotonic transducer (T2 bioscience instruments, UK). Tyrode’s solution was oxygenated during the study and its temperature was maintained at 37 ^o^C. Responses were recorded using an oscillograph device (the bioscience 400, UK). To evaluate the spasmolytic action of hops, after taking a baseline response, the specimens (n=6 for each hops extracts concentration) were first exposed to 10^-4^ M acetylcholine (Sigma Aldrich Chemie Gmbh, Germany) as a standard stimulant of gastrointestinal smooth muscle and then either to the different concentrations of hops extracts (0.1, 0.3, and 0.5 mg/ml), 10^-6^ M atropine sulphate (Sigma Aldrich Chemie Gmbh, Germany) or normal saline. The amplitude of contractions induced by cumulative 10^-12^ up to 10^-2^ M acetylcholine after 2 min tissue exposure to the same concentrations of hops extracts or normal saline (n=6), was assessed to evaluate the antispasmodic action. 

**Statistical analysis**

The effects of different solutions were measured as the change in the contraction amplitude and were expressed as the percentage of mean±SEM decline in contraction as compared with maximum effect of acetylcholine. All statistical analyses and comparisons were made using means of the ANOVA followed by Tukey's post-hoc test. The statistical significance was considered as p< 0.05.

## Results

**Figure1:**
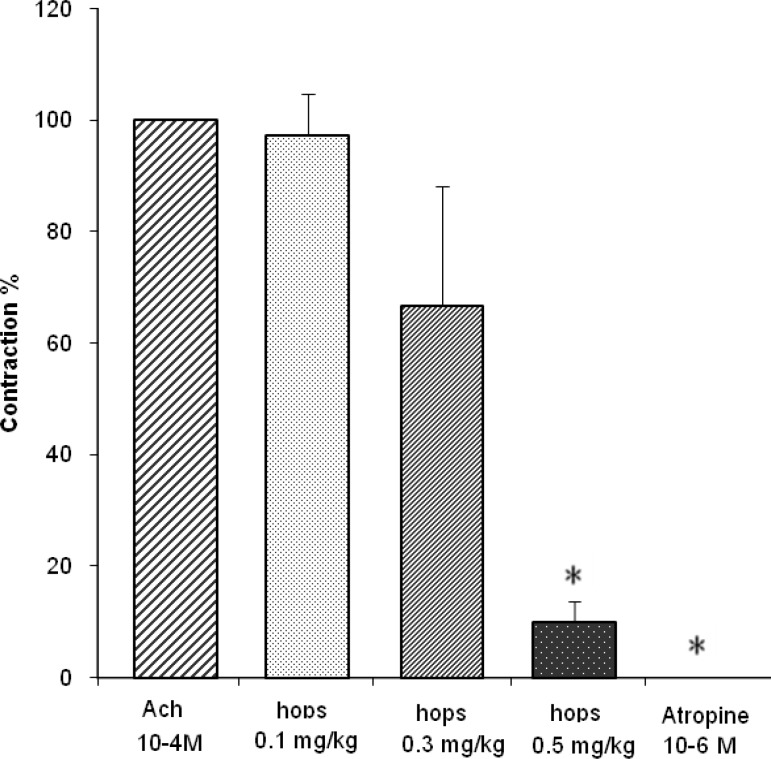
Spasmolytic effect of 10-6 M atropine sulfate and different concentrations of hops extracts on 10-4 M acetylcholine-induced contractions in isolated rat’s ileum (n=6). * indicates the significant difference (p<0.05) as compared with the Ach-induced contraction according to the one way ANOVA fallowed by Tukey’s post-hoc test.

The effective dose of Ach 10 ^-4^ M significantly elevated the baseline in all assessments (22.5±4.6 mm). All concentrations of hops extract had spasmolytic effect on acetylcholine-induced contraction in which the reduction in tonic contraction by 0.5 mg/ml hops extract was significant (p<0.05, Figure 1). 

**Figure 2:**
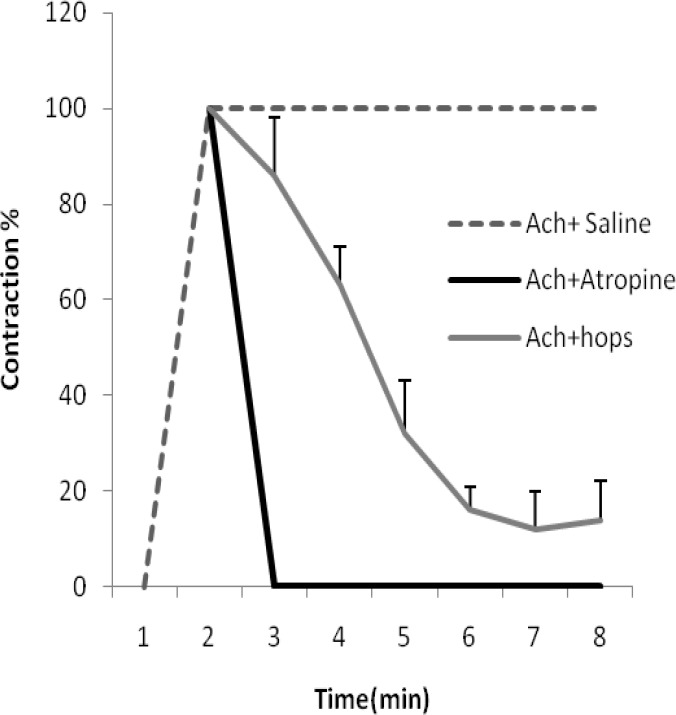
The time course inhibitory effect of atropine (10^-6^ M) and 0.5 mg/ml hops extracts on acetylcholine-induced contraction (n=6).

**Figure 3:**
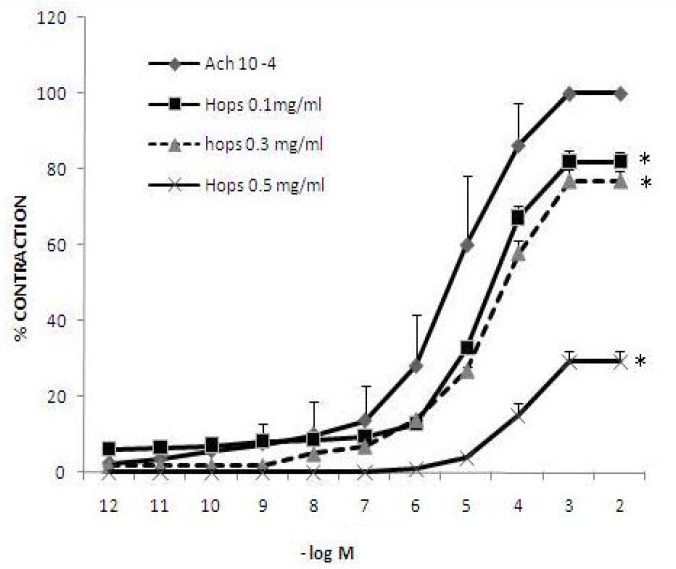
Antispasmodic effect of hops extracts on isolated rat’s ileum (n=6). * indicates the significant difference between the maximum contraction induced by 10^-3 ^M acetylcholine in the presence of saline and 3 different concentrations of hops extracts (p<0.05), according to the one way ANOVA fallowed by Tukey’s post-hoc test

The contractile effect of Ach was also significantly diminished by the infusion of 10^-6^ atropine sulfate. The inhibitory effect of atropine sulfate on Ach-induced contraction was observed after a few seconds and completed in 1 min, while the inhibition due to 0.5 mg/ml hops extracts was completed after 5 min and remained at about 10% of maximum contraction induced by Ach (Figure 2). In this case, the inhibitory effect of 10^-6^ atropine was significantly greater than 0.5 mg/ml hops extracts (p<0.05). Pre-exposing the tissues by 3 concentrations of hops extract have antispasmodic effect on amplitude of contraction induced by 10^-6 ^up to 10^-3 ^M Ach in a concentration-dependent manner (Figure 3). The effect of acetylcholine (10^-3 ^M) -induced contractions were significantly inhibited by 0.1, 0.3, and 0.5 mg/ml hops extract (81.9, 77, and 29.3%, respectively, p<0.05).

## Discussion

According to our findings, hops extract induced a relaxant effect on ileal smooth muscle. Different mechanisms are involved in gastrointestinal smooth muscle relaxation by plant extracts. These include the blocking action on muscarinic M_3_, histaminic H_1_ and 5-HT receptors (Natividad et al., 2011 ▶; Estrada-Soto et al., 2012 ▶). The relaxant effect may also be induced by stimulating the nitric oxide (Kurjak et al., 2011 ▶), purinergic (Van Crombruggen et al., 2007 ▶), adrenergic (Roberts et al., 1999 ▶), or GABAergic modulatory systems (Zizzo et al., 2007 ▶**)**. According to our literature review, there is no scientific document indicating the effects of hops on smooth muscle contraction. 

However, in 2005 Kurasawa et al. (Kurasawa et al., 2005 ▶) demonstrated an increase in the gastric secretion by hops extract similar to the carbachol action. They concluded that this stimulatory action may be induced via stimulating the enteric cholinergic neurons. Our findings that hops extract inhibited the intestinal motility contrast this suggestion since the cholinergic system is the most important excitatory transmitter regarding the intestinal smooth muscle contraction (Koniger et al., 2013 ▶). However, other studies reporting the effects of hops on sleeping and sedation (Dimpfel et al., 2008 ▶; Juanez, 2012 ▶; Ross, 2009 ▶) indicate that these actions are through inhibition of central nervous cholinergic system, which are in accordance with our findings indicating the inhibitory effect of hops extracts on Ach-induced contractions in a peripheral tissue. This action may be due to the blockade of muscarinic receptors on the ileal smooth muscle cells or via a competitive inhibitory effect on Ach-induced contractions. 

In the other hand, it has been demonstrated that hop aqueous extract attenuated allergic responses via its inhibitory action on the release of histamine and leukotrienes from the mast cells (Segawa et al., 2007 ▶). Therefore, part of the inhibitory action on ileal smooth muscle contraction expressed by the hops extract may be due to its antihistaminergic activity. Another probable mechanism which may inhibit the contraction of gastrointestinal smooth muscle is via activating the nitric oxide release from the enteric neurons (Kutchai, 2006 ▶**)** but Zhao et al. (Zhao et al., 2003 ▶) have reported a significant inhibition of inducible nitric oxide synthase and nitric oxide production induced by hops extract. Therefore, our finding that hops extracts could inhibit the rats ileal contraction, opposes these findings and indicates that the nitric oxide system (as an inhibitory system in ileum) may not be inhibited by hops extracts. It has been suggested that hop oil potentiates the GABA receptor sensitivity (Hitoshi et al., 2006 ▶**).**

**Therefore,** the spasmolytic effect of hops extract on rat’s ileum may be in part due to the activation of these receptors which are among the inhibitory mediators of intestinal smooth muscle contraction (Kawakami et al., 2004 ▶; Kanako et al., 2007 ▶). However, the involvement of histaminergic/GABAergic systems, calcium influx, or any other possible mechanisms in relaxant effects of hops extract on rat’s illeal contraction should be further investigated. 
